# Advances and Perspectives in Relation to the Molecular Basis of Diabetic Retinopathy—A Review

**DOI:** 10.3390/biomedicines11112951

**Published:** 2023-11-01

**Authors:** Michał Błaszkiewicz, Agata Walulik, Kamila Florek, Ignacy Górecki, Olga Sławatyniec, Krzysztof Gomułka

**Affiliations:** 1 Student Scientific Group of Adult Allergology, Wroclaw Medical University, 50-369 Wroclaw, Poland; 2 Clinical Department of Internal Medicine, Pneumology and Allergology, Wroclaw Medical University, 50-369 Wroclaw, Poland

**Keywords:** diabetes, proliferative diabetic retinopathy, retina, vascular endothelial growth factor, asymmetric dimethylarginine, microRNAs, endothelin-1, advanced glycation end products

## Abstract

Diabetes mellitus (DM) is a growing problem nowadays, and diabetic retinopathy (DR) is its predominant complication. Currently, DR diagnosis primarily relies on fundoscopic examination; however, novel biomarkers may facilitate that process and make it widely available. In this current review, we delve into the intricate roles of various factors and mechanisms in DR development, progression, prediction, and their association with therapeutic approaches linked to the underlying pathogenic pathways. Specifically, we focus on advanced glycation end products, vascular endothelial growth factor (VEGF), asymmetric dimethylarginine, endothelin-1, and the epigenetic regulation mediated by microRNAs (miRNAs) in the context of DR.

## 1. Aim

The study aimed to indicate the vital biomarkers and molecules involved in the pathomechanism of diabetic retinopathy, evaluate their impact, assess their diagnostic value for staging or risk assessment of DR, and specify the potential point of grasp for therapeutic methods.

## 2. Introduction

Diabetic retinopathy (DR) remains a major ocular complication of diabetes mellitus (DM) and is the leading cause of irreversible yet preventable vision loss among the working-age adult population, particularly in low- and middle-income areas [1]. Among the 537 million adults (20–79 years) with DM, approximately one-third have signs of DR, and one-third of this group may go on to develop severe retinopathy or macular edema [2,3]. Apart from ocular effects, the presence of DR also signifies an evaluated future risk of myocardial infarction, heart failure, and cerebrovascular accidents [4].

As the worldwide prevalence of diabetes mellitus has significantly increased over the past twenty years, the persistently high incidence of diabetic retinopathy among individuals with diabetes makes screening crucial. This screening is necessary for the early detection of individuals displaying signs of visual impairment due to chronic hyperglycemia, who require a comprehensive ophthalmic examination and appropriate treatment [5].

The COVID-19 pandemic had a significant impact on DR screening, monitoring, and treatment process. According to a study performed in 2020, the Cole Eye Institute, USA, the average delay in care for patients who missed their appointments during the pandemic was 5.34 weeks [6]. Another retrospective study of DR patients who attended the DR screening program at Hospital Universitari Sant Joan de Reus revealed that the number of screened patients decreased to 3286 (57.89%) in 2020, compared to an average 5676.40 ± 439.75 of screened patients between 2015 and 2019. In 2021, this number increased again, resulting in 6804 screened patients [7]. Centers for Disease Control and Prevention (CDC) reports that the prevalence of DR in 2019 in the USA was 3.11% of the USA population [8]. Whereas, 2021 screening programs reported by CDC resulted in a decrease of 2.89% of DR patients [8]. However, the COVID-19 pandemic accelerated the development of screening tools like digital ophthalmoscopes (DOs) for DR diagnosis. Those solutions can be used without prior extensive traineeship in that field by general practitioners or patients by themselves. Although the pandemic is over, there is a possibility to take advantage of using those telemedicine devices in areas with limited access to ophthalmologists. Nevertheless, there are no international guidelines concerning DO usage, but the inclusion of those techniques should be considered [9]. There are different DOs, desktop-based DOs, handheld DOs, and smartphone-based retinal imaging DOs, and their sensitivity ranged from 61% to 81% between different studies considered in the most recent systematic review [10]. However, the NO BLIND study showed 100% specificity and 94.3% sensitivity for digital ophthalmoscope usage by ophthalmology specialists as compared to standard fundus oculi examination in mydriasis [11]. The role of telemedicine in the field of DR should be highlighted as it is a cost-effective approach and it is a tool that enables broad screening and faster diagnosis making [9].

This article provides a summary of the current knowledge regarding diabetic retinopathy, especially emphasizing the molecules that could serve as markers of ongoing pathological processes in the retina.

## 3. Risk Factors

The most relevant risk factors for the development of diabetic retinopathy include the duration of diabetes, greater uncontrolled hyperglycemia as indicated by high HbA1c levels, and the presence of hypertension [12]. Research showed that maintaining proper blood glucose control has a notably stronger effect on DR prevention compared to controlling blood pressure [13,14]. Research suggests that the risk gradually increases over time, making regular eye examinations essential for individuals with diabetes identified for more than a decade.

Other well-known risk factors for DR are nephropathy and high BMI index [12,15]. Although there are no definite associations between traditional lipid markers and DR, several studies over the years have suggested that lipid-lowering therapy might be an effective adjunctive agent for DR and may reduce the risk of its development [16,17,18,19,20]. Both diabetic retinopathy and nephropathy are complications of DM resulting from microvascular damage through, i.e., inflammation and oxidative stress attributed to uncontrolled blood glucose levels. These mechanisms can result in the simultaneous occurrence of nephropathy and DM, consequently, it is important to regularly screen patients with severe nephropathy for eventual DR development [3,21].

Smoking is an additional risk factor for DR. Xiaoling et al., in the study identifying and comparing 73 studies involving type 1 and type 2 diabetes patients, established a clear association between smoking and DR. In type 1 diabetes, the risk of DR significantly increased among smokers compared to non-smokers. Surprisingly, in type 2 diabetes, the risk of DR was found to be lower in smokers than in non-smokers [22]. However, this result should not change the importance of smoking cessation for overall health benefits.

Research findings indicate that for women with diabetes, pregnancy can pose an additional risk factor for developing or worsening already existing DR. The prevalence of DR in women with type 1 diabetes is higher than in type 2 and it tends to worsen in type 1 diabetic women compared to type 2 [21,23]. Consequently, it is crucial for pregnant women with diabetes to closely monitor blood glucose levels and manage the condition effectively.

**Figure 1:**
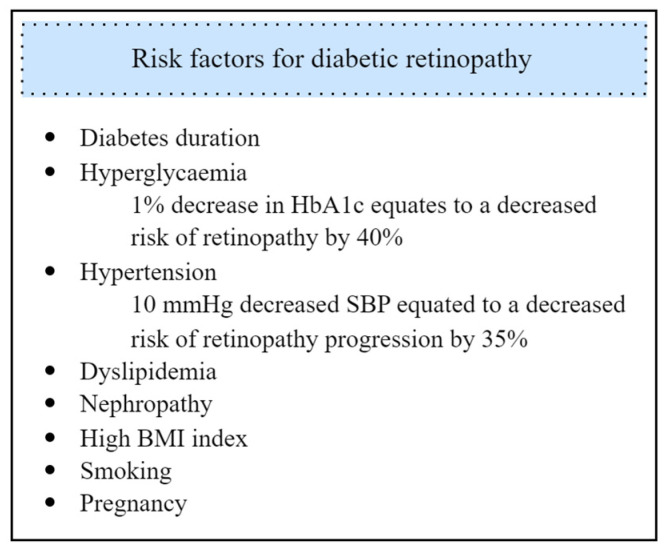
Risk factors for diabetic retinopathy development.

Diabetic retinopathy is a complex condition that requires diligent management to prevent or slow down its progression. By understanding the risk factors associated with diabetic retinopathy, presented in Figure 1, individuals with diabetes can take proactive measures to protect their vision. Consistently managing blood sugar levels, blood pressure, and cholesterol and making healthy lifestyle choices, such as quitting smoking, are crucial steps in reducing the risk and severity of diabetic retinopathy.

## 4. Pathophysiology

Diabetic retinopathy is primarily associated with microvascular abnormalities and retinal neurodegeneration [24]. The neurovascular unit comprises endothelial cells and pericytes, basement membrane, glial cells (including astrocytes and Müller cells), microglia, and neurons. The degeneration of this unit is considered a primary indicator of diabetic retinopathy [25].

Hyperglycemia induces non-enzymatic advanced glycation end products creation, increases oxidative stress, and promotes the growth in proinflammatory cytokines, leukocyte migration, and adhesion, which may lead to leukostasis (microcapillaries blockade with leukocytes), moreover, it influences epigenetic modifications [26,27]. Hyperglycemia, chronic inflammation, and microthrombi induce hypoxia and via hypoxia-inducible factor (HIF-1α) upregulates growth factors, mainly VEGF (vascular endothelial growth factor) [28]. The VEGF isoforms promote endothelial cell proliferation during early angiogenesis, and some of its isoforms take part in pathological neovascularization. Furthermore, VEGF increases vascular permeability by disrupting the tight-junction between retinal endothelial cells [29,30].

Hypertension and local retinal vasoconstriction also play a role in DR development and are associated with increased VEGF production [31].

The clinical classification divides DR into non-proliferative diabetic retinopathy (NPDR) and proliferative diabetic retinopathy (PDR) [32]. In the pathogenesis of NPDR, there is a loss of pericytes, a decrease in their protective role, damage to endothelial cells, and excessive thickening of the basement membrane. These changes lead to vascular leakage and cellular damage [28,33]. The clinical manifestation of NPDR in the fundoscopic examination typically reveals microaneurysms, which may rupture and cause hemorrhages. Additionally, vascular leakage can result in the appearance of hard exudates. Macular edema can also occur, and due to the occlusion of microcapillaries, ischemia may develop, leading to nerve fiber infarcts that are visible as “cotton-wool spots” [28]. PDR can occur as a progression from NPDR, but it can also manifest without a preceding NPDR stage. This severe clinical presentation is associated with pathological neovascularization and fibrous proliferation, manifesting as epiretinal or vitreous hemorrhages causing temporal vision loss and retinal detachment leading to permanent blindness [28,34]. Macular edema can be a cause of both NPDR and PDR and is considered reversible damage [35].

## 5. Molecular Biomarkers

In recent years, the exploration of biomarkers has gained significant momentum, offering novel insights into the pathogenesis and progression of diabetic retinopathy. Biomarkers are measurable biological indicators that reflect normal or pathological processes within the body. They can be used to diagnose, stage, characterize, and monitor diseases, individualize therapeutic interventions, and monitor responses to the therapies [36]. They can be detected in various bodily fluids, such as blood, tears, or even ocular tissues, offering a non-invasive and precise means of diagnosis and monitoring. This article delves into the topic of possible biomarkers in diabetic retinopathy, shedding light on the advancements, challenges, and prospects of these innovative diagnostic tools. We will explore the potential role of biomarkers in detecting the disease at its earliest stages, predicting its progression and tailoring personalized treatment strategies.

### 5.1. Vascular Endothelial Growth Factor

**Figure 2:**
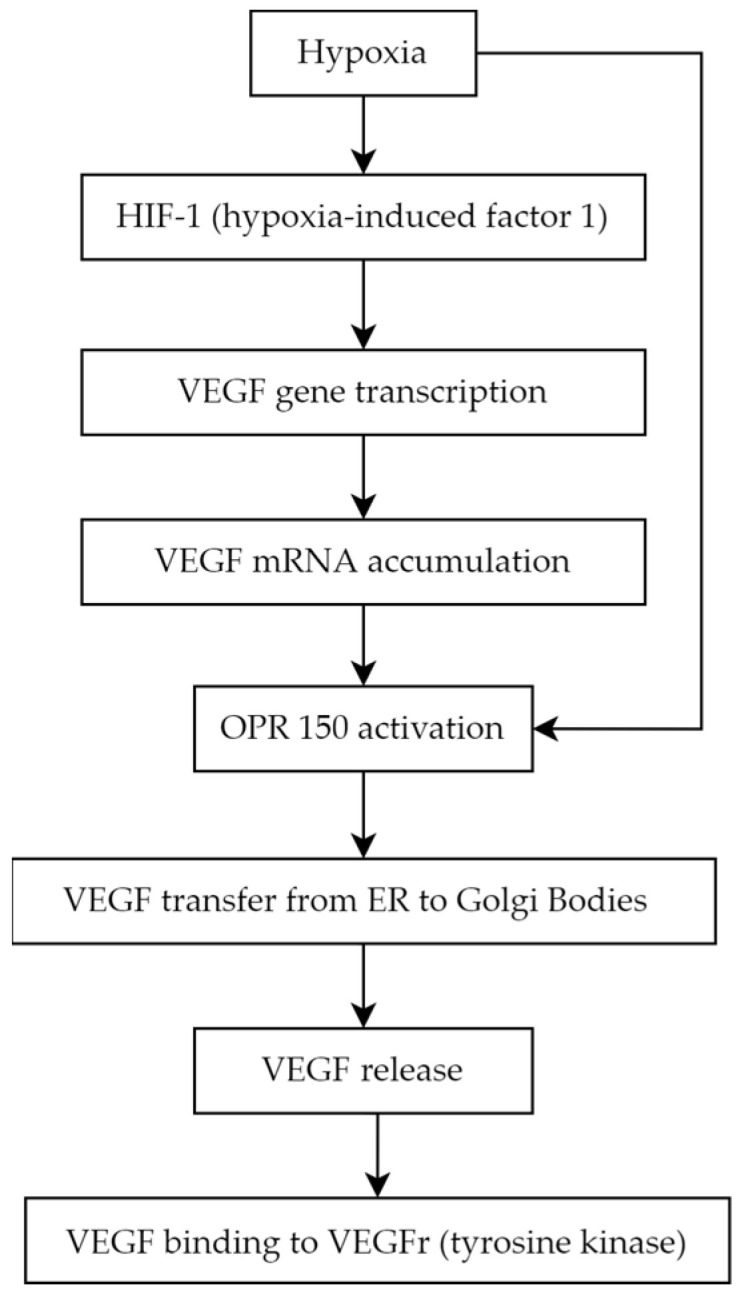
VEGF production and secretion pathway.

Angiogenesis is a complex biological process that underlies the development of proliferative diabetic retinopathy, which represents the advanced stage of diabetic retinopathy [37]. Among the many pro-angiogenic factors, vascular endothelial growth factor (VEGF) is notably significant. VEGF is a homodimer glycoprotein with a molecular weight of 46 kDa connected by three disulphide bonds (cystine-knot form). The VEGF family consists of the following members: VEGF-A (also called VEGF or vascular permeability factor as the first discovered molecule of the whole family in 1983), VEGF-B, VEGF-C (essential for the formation of lymphatic vessels) [38], VEGF-D (known as c-Fos-induced growth factor, FIGF), VEGF-E (connected with parapoxvirus Orf, which causes pustular dermatitis) [39], and placenta growth factor (PGF) molecules [40,41]. VEGF production is stimulated by ischemia and hypoxia. Low pO_2_ induces the production of the crucial mediator of hypoxic responses—DNA-binding protein called hypoxia-induced factor 1 (HIF-1). HIF-1 binds to specific enhancer elements, which stimulates the transcription of the VEGF gene and, in turn, VEGF mRNA production and decreased mRNA degradation. Accumulated intracellular VEGF is transported from endoplasmic reticulum to Golgi bodies by a chaperone protein known as ORP 150 (oxygen-regulated protein 150), whose secretion is augmented in a hypoxic environment [42,43] (Figure 2).

VEGF has been shown to play a role in physiological processes such as vasculogenesis—de novo formation of blood vessels during embryogenesis, angiogenesis—vessels formation from already existing vasculature, and pathological processes like tumor growth, tissue remodeling, and metastasis. VEGF mediates its effects by binding to the tyrosine kinase receptors (VEGFRs). The family of VEGFRs consists of the following members: VEGFR1 and VEGFR2 (mainly located on blood vessels endothelial cells) and VEGFR3 (expressed in lymphatic endothelium) (Table 1). The molecular structure of each receptor is similar [44].

VEGFR1 can be secreted in the soluble form (sFlt1—expressed in the placenta during gestation) to prevent endothelial overproliferation due to VEGF accumulation. There are known VEGF coreceptors as well: the neuropilins, neuropilin-1 and neuropilin-2. Their main role is to enhance the binding of VEGF to the VEGFR2 and the migration of endothelial cells stimulated by VEGF [45]. Heparan sulfate and integrins can also modulate the signal from VEGFR. VEGFR1 is a crucial factor in providing a proper level of VEGF-A, and it plays an important role in the negative regulation of vascular modification. VEGFR1 is also necessary for the process of monocyte migration. PGF bound to VEGFR1 is responsible for initiating inflammatory-related angiogenesis, which is vital in the pathogenesis of various diseases. VEGF-B characteristically binds to VEGFR1 in tissues with high metabolic activity such as myocardial cells. VEGFR2, the most comprehensively studied among the VEGF receptors, binds with VEGF-A, VEGF-C, and VEGF-D. It is known that the activation of VEGFR2 kinase through VEGF-induced VEGFR2 homodimerization is responsible for the majority, if not all, of the known VEGF-related processes such as mitosis stimulation, migration, and survival of endothelial cells, ultimately leading to the formation of new blood vessels [46]. The crucial functions of VEGFR2 in endothelial biology are evident from the various processes involved in its tight regulation such as internalization to early endosomes for the activation of specific pathways and the involvement of phosphatases like vascular endothelial protein tyrosine phosphatase. VEGFR3 has an affinity to VEGF-C and VEGF-D. Although the main function of VEGFR3 was initially identified as the regulation of lymphatic endothelial development and biology, it has also been observed to be present in blood vascular endothelial cells. Angiogenesis-involved endothelial cells exhibit the expression of both VEGFR2 and VEGFR3. VEGFR3 activation is not solely dependent on VEGF-C/VEGF-D binding, as it can also be activated through integrin-mediated mechanisms, it leads to lymphatic vessel expansion and the absorption of interstitial fluid [47]. In addition, VEGF is critical for ensuring the proper morphology and function of vascular structures [46]. Notably, mutations that result in the loss of the VEGF gene allele have been found to be lethal [48]. The development of PDR is linked to relative retinal ischemia, which creates a hypoxic environment, which favors HIF-1 activation and VEGF production. The pathological revascularization in the retina has been attributed to VEGF-A165, a specific splice variant of VEGF-A [49].

As opposed to levels of plasma VEGF, elevated intraocular VEGF has been strongly associated with macular edema and retinal angiogenesis [50]. It has been proven that the aqueous VEGF level is correlated with this in the vitreous [51,52]. What is more, current research revealed that in DR patients, vitreous and aqueous VEGF levels are significantly higher than the plasma levels and they are both associated with DR progression [52]. However, some studies show that the median serum VEGF levels are found to be greater in patients with DM, PDR, and NPDR than in the matched control groups [53,54]. Furthermore, the serum VEGF levels are significantly higher in PDR than in NPDR patients [55]. According to current studies, serum VEGF seems to be an appropriate biomarker for DR onset and severity as well [55,56]. Nonetheless, changes in circulating VEGF-A levels cannot be used as a predictive factor of DR progression [50,53]. Moreover, the increase in serum PGF levels has been observed in NPDR patients treated with aflibercept (anti-VEGF antibody). In PDR patients who underwent the same therapy, PGF remained stable. Increased PGF levels may result from a counter-regulatory mechanism, due to VEGFR2 inhibition. However, in PDR patients, permanent uncontrolled expression of VEGF probably masks the counter-regulated PGF secretion [53]. Current studies reveal that the tear VEGF level is elevated in patients with DR and substantially different between NPDR and PDR patients. What is more, the VEGF level in tears is significantly associated with DR stage and severity [57,58,59]. There are studies presenting useful methods for tear VEGF level measurements characterized by high sensitivity and specificity [60,61]. Recent studies indicate the importance of anti-VEGF therapy, especially intraocular injections of those drugs [62].

### 5.2. Asymmetric Dimethylarginine

Arginine is an amino acid essential for normal growth and development. Endogenous synthesis is adequate in healthy people but might be deficient in many pathological states [63]. The earliest sign of vascular complications is endothelial dysfunction [64]. Nitric oxide (NO) is an important vasodilator that is crucial in maintaining the health of the vascular endothelium. Studies demonstrate that endothelial dysfunction plays a critical role in the development of diabetes-associated microvascular complications and often precedes advanced diabetic retinopathy (DR) [65,66,67]. NO is synthesized from the guanidine group of arginine by the enzyme family NO synthases (NOSs), which consist of three isoforms [68,69]. Asymmetric dimethylarginine (ADMA) is an active endogenous methylated amino acid, a structural analogue of L-arginine, which inhibits the activity of all isoforms of NOS, inhibiting the formation of nitric oxide in tissues and blood plasma [70,71]. ADMA is synthesized by the protein arginine N-methyltransferase 1 (PRMT1), mainly metabolized by the dimethylarginine dimethylaminohydrolases (DDAHs) pathway, and eliminated from the body by kidneys [72,73]. ADMA enters cells through cationic amino-acid transporters (CATs) [74]. Plasma levels of ADMA in healthy people vary between 0.3 and 0.5 μmol/L [75], but in pathological states, it may increase even tenfold [76]. ADMA has a negative effect on cells, contributing to oxidative stress, shortening telomeres, inhibiting the release of NO, and increasing the secretion of interleukin-8 and monocyte chemotaxis factor 1 [75]. Under normal conditions, endothelial NOS is inhibited by 10%, but in pathological situations, even by 30–70% [76]. When the plasma ADMA level increases, the NO synthesis in the environment decreases, vascular homeostasis degrades due to vasoconstriction, and endothelial dysfunction begins [69]. Endothelial dysfunction and impaired ocular hemodynamics prime diabetic retinopathy development are associated with decreased NOS activity and NO bioavailability, thus resulting in increased reactive oxygen species (ROS) and vasoconstriction [76,77]. Oxidative stress is closely related to DDAH activity, which further affects ADMA concentrations in patients with diabetes [78,79]. Increased oxidative stress contributes to elevated ADMA, and by the upregulation of circulating markers of oxidative stress, increased serum ADMA concentration is associated with increased vascular oxidative stress [80,81,82]. ADMA accumulation was first reported in patients characterized by endothelial dysfunction including hyperglycemia, hypercholesterolemia, and hypertension [83,84]. Impaired liver or renal function could also have an impact on the plasma concentration of ADMA. The significance of ADMA in the inhibition of vascular endothelial growth factor-mediated angiogenesis has been demonstrated in numerous studies. Some evidence suggests that diabetes mellitus with microvascular complications has increased serum levels of ADMA [85,86,87,88]. Elevated ADMA was detected in aqueous humor in diabetic patients, especially those with severe retinopathy [89]. The plasma ADMA level is elevated in patients with diabetic microangiopathy such as DR [66,86,90,91,92,93]. Lowering ADMA levels may delay the progression of DR by reducing the formation of neovascularization, providing protective advantages for the blood–retinal barrier [92]. Some clinical studies have shown that ADMA levels in diabetic patients with retinopathy were higher than among individuals without retinopathy, and this increase was directly proportional to the severity of diabetes [65,94,95,96]. High levels of ADMA have been identified not only in advanced DR but also in individuals at the prediabetic and diabetic stages. This observation suggests that ADMA likely plays a crucial role in both the initiation and advancement of DR [81,97]. Further studies that involve larger patient populations to better understand the role of plasma ADMA levels in the development and progression of DR are needed. The serum ADMA level was accepted as a marker of endothelial dysfunction because of its high values in coronary artery disease, end-stage renal failure, stroke, hypertension, and DM [98,99,100,101]. In the present moment, there are no drugs targeting ADMA levels in the context of diabetes management [102].

### 5.3. MicroRNAs

MicroRNAs (miRNAs) are single-strengthened, non-coding RNA, which affect gene expression regulation. Their suppressor interaction with mRNA usually is associated with 3′ untranslated regions (3′ UTRs), although data claim as well its interaction potential according to different sequences such as gene promoters. Moreover, they also have a regulatory role in transcription and translation processes [103]. The creation process of those micromolecules goes from DNA transcription to primary miRNA (pri-mRNA) through precursor miRNA (pre-miRNA) leading to mature miRNA formation [104]. The role of miRNA in signalization pathways is studied nowadays excessively because of those particles’ multiplicity. Furthermore, their remarkable stability in circulation makes them attractive potential non-invasive biomarkers and therapeutic grip points [105]. Their role as a biomarker may be a useful tool in the DR diagnostic process because currently assessment is based only on ophthalmological examination and the inclusion of non-invasive objective tests into guidelines could enhance DR detectability.

Molecular bases of miRNA mechanisms of action are distinct for different miRNAs, and it is possible to distinguish which particles affect which pathway leading to DR, such as affecting cell proliferation, angiogenesis, apoptosis, or basement membrane thickening [106]. It has been proven that directly or indirectly particles such as miRNA-9, miRNA-152, miRNA-15b, miRNA-29b-3p, miRNA-199a-3p, miRNA-203a-3p, miRNA-200b-3p, and miRNA-30a-3p downregulate VEGF expression, which lowers the range of active cell-cycle-related proteins and by that protects RMECs (retinal microvascular endothelial cells) from abnormal proliferation [107]. In addition, from previously mentioned biomolecules, the alternative pathway to downregulate VEGF is SIRT1 (nicotinamide adenosine dinucleotide (NAD+)-dependent deacetylase) upregulation, which is possible by miRNA-29b-3p and miRNA-34a inhibition, moreover, causing an increase in proinflammatory cytokines [107]. MiRNA-34a was evaluated to be an interesting therapeutic target, as in rats with induced DR, its silencing was observed as an apoptosis regulation [108]. Relatedly, the miRNA overexpressed in DR upregulating SIRT1 is miRNA-195, which inhibits RMEC and accelerates apoptosis [109]. In addition, miRNA-210 was assessed as a potential biomarker able to distinguish PDR from NPDR; moreover, it refers to DR severity, and progression as well is considered a therapeutic target according to vascular endothelial cells pathological proliferation [110].

**Figure 3:**
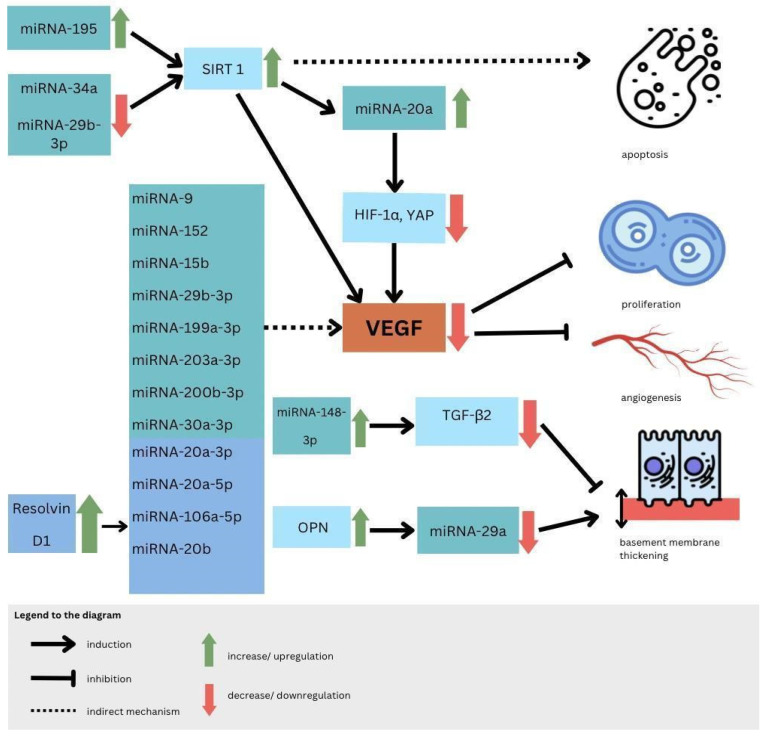
MiRNA impact on apoptosis, angiogenesis, proliferation, and basement membrane thickening in DR.

MiRNA-20a and miRNA-20b were revealed to downregulate VEGF as well but in different mechanisms—first act by Yse-associated protein (YAP)/hypoxia-inducible factor 1α (HIF1α)/VEGF axis, and second was revealed in the study on rats to be correlated with downregulation of AKT3, lowering VEGF expression [111,112]. Moreover, it was assessed that Resolvin D1 modulates the intracellular VEGF-related miRNAs—miRNA-20a-3p, miRNA-20a-5p, miRNA-106a-5p, and miRNA-20b—expression of retinal photoreceptors challenged with high glucose [113]. Another pathway included in DR pathogenesis and connected to miRNA function is the thickening of basement membrane by increased synthesis of RMEC extracellular matrix—especially the abnormal synthesis of collagen type 4 due to miRNA-29a inhibition by inflammatory factor OPN (osteopontin) also known as secreted phospho-protein 1 (SPP1) [114]. It is needed to mention that there was a confirmed association between OPN and vascular hyperpermeability in human diabetic retinal tissues, and by a blockade of OPN, vascular continuity was preserved, in contrast, by the miRNA-148a-3p action-TGFβ̞2 expression was lowered and by that basement membrane thickening was decreased [115,116] (Figure 3).

The role of the miRNA was investigated as a DR biomarker using different sample types and designs compared to various groups according to diabetes type 1 or 2, T1DM or T2DM, patients with DM and healthy individuals, as well studies referring to DR progression. In blood serum samples in T1DM patients with DR and those without retinopathy, the most significant was miRNA-211. Then, miRNA-18b and miRNA-19b were revealed as upregulated; additionally, miRNA-29a, miRNA-148a, miRNA-181a, and miRNA-200a were revealed to have such an impact [117,118]. Furthermore, miRNA-93, miRNA-21, and miRNA-146a are downregulated in T1DM patients [119]. Trials which evaluated biomarkers correlation with DR progression among T1DM revealed as the most important factors certain macromolecules, for PREVENT-1-higher, miRNA-320a concentration, and in PROTECT-1-lower, miRNA-27b expression [120].

According to T2DM, a study was performed and the differences in the following particles were noted: hsa-let-7a-5p, hsa-miRNA-novel-chr5_15976, hsa-miRNA-28-3p, hsa-miRNA-151a-5p, and hsa-miRNA-148a-3p were upregulated compared to DM group with no retinopathy; however, a panel of the first three of them were the closest to help in assessing the diagnosis as its sensitivity and specificity were as follows: 0.92 and 0.94 [121]. Another study showed that in T2DM patients, DR was associated with increased circulating levels of miRNA-25-3p and miRNA-320b and decreased levels of miRNA-495-3p [122]. In addition, it was revealed in the study performed on rats with induced diabetes that miRNA-200b in DR diseases is lower than in the healthy group [123]. According to the vitreous humor, there were studies suggesting changes in several particle concentrations in PDR groups: miRNA-125, miRNA-21, hsa-miRNA-6734-5p, and hsa-miRNA-1297 [124,125]. MiRNAs, such as hsa-miRNA-3184-3p, hsa-miRNA-24-3p, and hsa-miRNA-197-3p, were assessed to be upregulated in the vitreous humor of PDR patients; furthermore, anti-VEGF-factor administration led to lower expression of those particles [124]. However, vitreous humor parameter measurements are not easy to perform.

**Figure 4:**
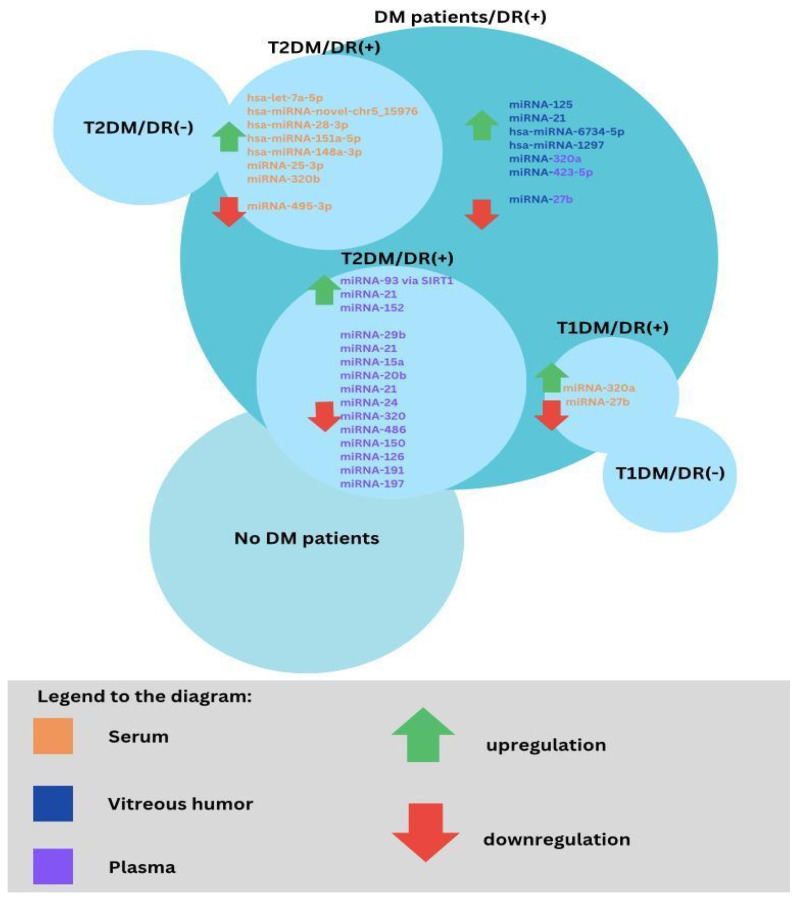
Changes in miRNA regulation depending on DM type and presence of DR in specific sample types. Overlapping circles constitute the study and control groups, while the increase or decrease in regulation of miRNAs was marked only in the study group.

Plasma results among T2DM patients gave an insight into lower levels of miRNA-29b in the DR group and miRNA-21 as biomarkers that were significantly associated with PDR. Other parameters that were increased in T2DM patients with DR were miRNA-93 via SIRT1 and miRNA-21, as well as miRNA-152 [126,127]. On the contrary, miRNA-15a, miRNA-20b, miRNA-21, miRNA-24, miRNA-320, miRNA-486, and miRNA-150, miRNA-126, miRNA-191, miRNA-197 are downregulated in that group of patients’ plasma samples [128]. Importantly, miRNA-150 is observed in both T1DM and T2DM patients’ circulation and in the neutral retina. That factor by Elk1 upregulation stimulates proinflammatory, pro-angiogenic, and apoptotic influences. Otherwise, a lower range of miRNA-150 in serum impacts Elk1 and Myb overexpression, resulting in the same as the previously mentioned pathway in microvascular complications and neovascularization leading to DR; so, according to that analysis, it is not only a diagnostic biomarker but as well is significantly involved in DR pathogenesis [129]. Importantly, there were two meta-analyses performed according to miRNAs role in DR diagnosis, which revealed its significance and utility. The first study included eight trials with 93 parameters and even though six miRNAs were consistently reported in at least two studies and in the same direction, after stratification by the type of biological samples, miRNA-320a and miRNA-423-5p were consistently reported to be upregulated in two studies using serum samples and two studies using vitreous humor samples, respectively. It was consistently shown that miRNA-27b was downregulated in two experiments using serum samples [130]. However, most recently, analysis assessed miRNA panels’ superior diagnostic value to single parameters results [131]. Furthermore, another result is the fact that miRNA-21 in five studies was revealed to be a useful tool in the diagnosis of DR and an early predictor of reactive oxygen species-mediated damage among patients at high risk for diabetes [130,131] (Figure 4).

### 5.4. Endothelin-1

Endothelin-1 (ET-1) in its active form is a 21-amino acid hormone that helps to maintain basal vascular tone and metabolic function in healthy individuals [132]. ET-1 is an endothelium-derived factor with proliferative, profibrotic, and proinflammatory properties [133], and it is the most abundantly expressed member of the endothelin family of proteins (ET-1, ET-2, and ET-3). Immature ET-1 undergoes extensive post-transcriptional processing that concludes with cleavage by endothelin converting enzymes (ECEs) and subsequent release of mature ET-1 primarily toward the interstitial space, and in smaller proportion, into the circulation [132]. ET-1 works on two different ET-1 receptor subtypes, ETA and ETB, to produce its various biological effects [134]. The first subtype, ETA receptors, is predominantly localized on vascular smooth muscle cells (VSMCs) of blood vessels where they mediate contractile and proliferative response to ET-1, whereas ETB receptors have a more composite relation to vascular regulation. ETB receptors can lead to vasodilation via the release of relaxing factors if they are present on endothelial cells or vasoconstriction when they are located on VSMCs in certain vascular beds [133]. Therefore, the overall effect of ET-1 on different tissues is largely dependent on the expression and relative densities of individual receptor subtypes. ET-1 is one of the important markers of endothelial dysfunction, a state characterized by disturbed balance between vasoconstrictors and vasodilators [135]. Due to its vasoconstrictive properties, ET-1 has been widely studied in terms of its role in hypertension and proved clinically significant, e.g., with the use of endothelin receptor antagonists for the treatment of patients with pulmonary arterial hypertension [136]. The vasoconstrictive and in turn hypertensive properties of ET-1 can explain a possible link between elevated plasma ET-1 level and retinopathy under ischemia, a finding relevant to diabetic retinopathy, which is thought to be the consequence of retinal ischemia. Animal models have shown that administration of ET-1 into the posterior vitreous body or the optic nerve leads to physiological and cellular damages of ischemic origin, including obstruction of retinal blood flow, elevated scotopic b-wave in electroretinogram, and apoptosis of cells in ganglion cell layer of the retina [137]. Moreover, in the retina, ET-1 and its ETA receptor have been shown to mediate decreased retinal blood flow during hyperglycemia and in DR. Chen et al. described that hyperglycemia augments ET-1-induced constriction of human retinal venules by activation of ETA receptors [138]. A direct link has been established between hyperglycemia and increased ET-1 secretion from endothelial cells [139]. Various studies demonstrated elevated plasma ET-1 in patients with type 1 or type 2 diabetes [133]. Additionally, patients with advanced DR appear to exhibit elevated ET-1 concentrations in the aqueous of the eye compared to those with early DR and the control group, with exact concentrations varying among individuals, although the severity of DR seems to be a major factor relating to baseline aqueous ET-1 levels [140]. Another study measuring aqueous humor ET-1 found higher concentrations of ET-1 and reduced total retinal blood flow in subjects with early non-proliferative diabetic retinopathy than in age-matched controls [141]. These findings support the claim that ET-1 dysregulation may contribute to the pathogenesis of DR, though aqueous humor ET-1 is still not an exhaustively studied topic. Ottoson-Seeberger et al. demonstrated that exogenous ET-1 causes peripheral insulin resistance in healthy humans [142]. Insulin itself can regulate vascular tone through the increase in ET-1 synthesis and release among other mechanisms [143]. Strong evidence regarding the role of ET-1 in the pathogenesis of diabetic microangiopathy showcases the potential of endothelin receptor antagonists in the treatment of DR. Studies on animal models support this presumption. The use of endothelin receptor A antagonist, atrasentan, in streptozotocin-induced diabetic mice showed attenuation of microvascular changes in the retina [144]. Chou et al. also examined the effects of atrasentan on diabetic mice with similar results, additionally noting significantly reduced retinal pericyte loss [145]. The application of endothelin receptor antagonists via intravitreal administration showed decreased vascular leakage and expression of VEGF and inflammatory factors [146]. Topical administration of bosentan, a dual endothelin receptor antagonist, appeared to prevent neurodegeneration induced by diabetes in mice by blocking and downregulating ETB receptors and shows a viable alternative route to oral administration [147]. There is a clear limitation to extrapolating results of animal studies to human subjects, and more investigation needs to be conducted to fully understand the potential of using ET-1 receptor antagonists as novel therapeutic agents in the treatment of DR. 

### 5.5. Advanced Glycation End Products

**Figure 5:**
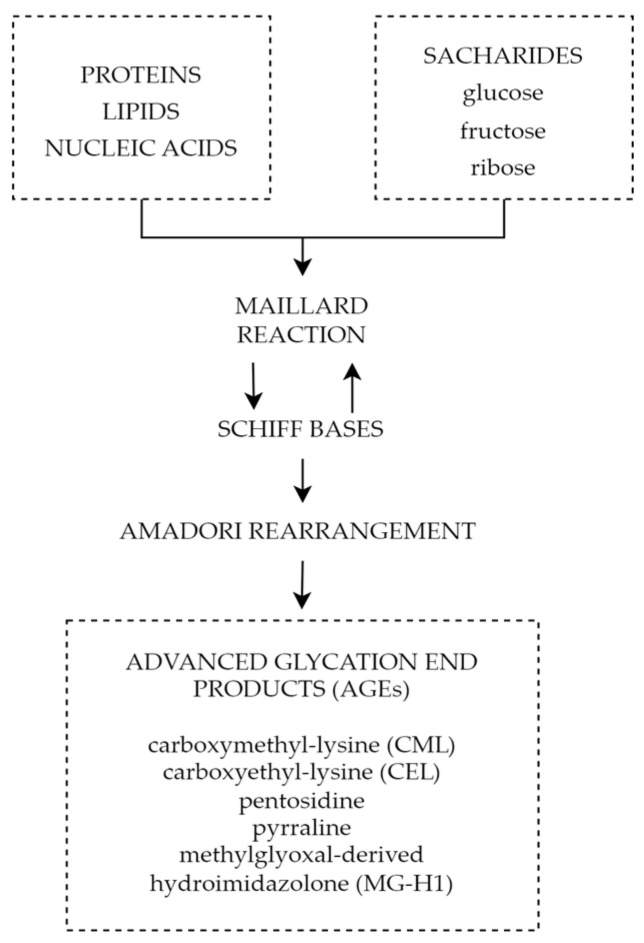
Forming of AGEs through Maillard reaction.

One of the mechanisms connecting chronic hyperglycemia with diabetic retinopathy is the formation and accumulation of advanced glycation end products (AGEs). Advanced glycation end products are heterogeneous groups of molecules formed from post-translational non-enzymatic modifications of proteins, lipids, or nucleic acids by saccharides including glucose, fructose, and pentose through the Maillard reaction represented by Figure 5 [148,149]. There are over 20 AGEs identified in human tissues, but some of the most common ones are carboxymethyl-lysine (CML), carboxyethyl-lysine (CEL), pentosidine, pyrraline, and methylglyoxal-derived hydroimidazolone (MG-H1) [150]. The characteristic factor of AGEs that distinguishes them from early glycation products, such as glycohemoglobin A1c (HbA1c), is the lack of spontaneous reversion ability, which once derived results in the accumulation in tissues over time [151]. Even though the discovery of AGEs dates to the early 20th century, not until the 1980s, the role of AGEs in aging and chronic diseases was recognized [152]. The first mention of AGEs and their accumulation in human tissues and their potential role in diabetic complications appeared in 1988 in a scientific article published by Helen Vlassara et al. [153]. Since then, AGEs and their involvement in pathophysiological processes have been the subject of extensive research. 

Excessive accumulation of AGEs in tissues has been found in aging processes and various chronic oxidative-based diseases, such as cardiovascular disease, neurodegenerative disorders, chronic renal failure, and most importantly in light of the following considerations, diabetes mellitus [154,155]. While in normal physiological conditions, the production of AGEs is controlled and moderate; however, under persistent hyperglycemia in diabetic patients, AGEs serum levels are much higher compared to the normal non-diabetic population. However, some endogenous factors such as oxidative stress and inflammation can also contribute to AGE formation [156]. AGEs accumulation may be as well through exogenous environmental and dietary sources. Only about 10–30% of AGEs present in food are fully absorbed through gastrointestinal mechanisms [157]. Many foods (e.g., red meat, aged cheeses, highly processed, packaged foods, and food with added sugars) contribute to increased AGEs levels in the body [158,159]. Besides, UV light, ionizing radiation, and air pollution induce high AGE production [160]. Advanced glycation end products play a significant role in the pathophysiology of diabetic retinopathy, one of the most common complications of diabetes mellitus. Even though the precise pathomechanism of its impact on the retina has not been already determined, it is associated with impairment of the neurovascular units (NVUs) through reactive oxygen species, inflammatory reactions, and cell death pathways [161]. AGEs can promote oxidative stress in retinal cells through binding with the receptor for AGEs (RAGE)—ubiquitously expressed in various retinal cells, including endothelial cells, pericytes, and neurons—leading to activation of various pro-oxidant and proinflammatory signaling pathways. A couple of major RAGE signaling pathways have been identified that can start key signaling cellular cascades to various ligands: mitogen-activated protein kinases (MAPKs) including p44/42 (ERK1/2), p38, and c-Jun N-terminal kinases (JNK); Janus kinase (JAK), signal transducer and activator of transcription (STAT); Ras-Rac-Cdc42; and phosphoinositide 3-kinase (P13-K)-Akt/PKB. Activation of the above mechanisms can induce DNA-binding activity among nuclear transcription factors such as STAT1/STAT3/STAT5, nuclear factor κB (NF-κB), and activator protein 1 (AP-1). RAGE under the condition of persistent hyperglycemia is mostly expressed in Müller cells—the ones highly susceptible to damage during DR. AGEs activate Müller cells, subsequently leading to increased production of VEGF, responsible for neovascularization, production of inflammatory cytokines, and monocyte chemoattractant protein-1 (MCP-1) [162,163,164,165]. Increased levels of AGEs lead to another pathological pathway: cross-link formation with proteins, resulting in the reduction in energy production, activation of endoplasmic reticulum (ER) stress, and macrophage activation [165]. AGE molecules have also been linked to the loss of pericytes and blood–retinal barrier (BRB) breakdown. The exact understanding of the mechanisms induced by AGEs in the pathophysiology of diabetic retinopathy is crucial for finding an effective treatment for this condition and should be the subject of future research. Analytical methods for the identification of AGEs include both instrumental and immunochemical methods. Spectrofluorometer, high-performance liquid chromatography coupled with mass spectrometry (HPLC/MS), gas chromatography coupled with mass spectrometry (GC–MS), liquid chromatography coupled with tandem mass spectrometry (LC–MS/MS), HPLC with fluorescent detection, and ultra-high-pressure liquid chromatography (UHPLC) are instrumental methods used to detect AGEs. Immunochemical methods contain enzyme-linked immunosorbent assay (ELISA) and Western blotting [166]. The measurement method is based on their chemical structure and ability to emit fluorescence. There are four groups as follows: (1) fluorescent and cross-linked (e.g., pentosidine); (2) fluorescent non-cross-linked; (3) non-fluorescent protein cross-linked; and (4) non-fluorescent and non-cross-linked (CML, CEL, pyrraline). These are especially important in vivo and may be used as biomarkers of pathological conditions such as diabetes and its complications. CML is frequently used as the AGE marker [160,167]. In recent years, research showed that it is more reliable to measure AGEs accumulation in accessible tissue using non-invasive methods than interpret their serum concentration since it does not necessarily reflect tissue AGE levels and depends on the half-time of the molecules [168]. The best choice for an AGE measurement method seems to be skin or lens autofluorescence since the lens crystallins and skin collagen are long-lived proteins reflecting long-term hyperglycemia exposure. Meerwaldt et al. in their research using the Autofluorescent Reader, measured the fluorescence of the skin in patients with type 1 and type 2 diabetes, correlating results with skin biopsies [169]. Autofluorescence clearly correlated with CEL, CML, and pentosidine levels with values higher than in control non-diabetic subjects. For comparison, Gerrits et al. during over 3 years of follow-up research involving 973 type 2 diabetic patients showed that skin autofluorescence was significantly higher in patients with microvascular, neuropathy complications developed but did not have predictive value for those with DR [170]. In the research of Osawa et al. involving patients with type 2 diabetic patients, skin AF was significantly increased with neuropathy, nephropathy, and diabetic retinopathy unlike the patients in the control non-diabetic group [171]. These findings suggest that skin autofluorescence may be a useful tool in identifying patients who are at risk of developing diabetic retinopathy. By measuring skin autofluorescence, it might be possible to identify patients with higher levels of AGEs—biomarkers of diabetes retinopathy and who may be at a higher risk of developing serious optic complications of persistent hyperglycemia. A deep understanding of the mechanisms leading to diabetic retinopathy through AGEs involvement completed via appropriate diagnostics could provide disease diagnosis, prognosis prediction, and therapeutic strategies. 

## 6. Discussion

Diabetic retinopathy is a crucial ocular complication of diabetes mellitus and the major cause of vision deterioration among the working-age adult population, particularly in low- and middle-income areas. Many factors, particularly the duration of diabetes, adversely affect the development and progression of DR. In recent years, interest in identifying factors that contribute to the development of diabetic retinopathy has increased significantly, as well as a better understanding of their role and mechanisms in its further progression. Moreover, the COVID-19 pandemic had a profound impact on the diagnostic and screening protocols for patients with diabetic retinopathy (DR). The restrictions imposed during the pandemic presented considerable challenges for both patients and healthcare professionals. These challenges compelled us to innovate and develop novel telemedicine solutions. Even though the COVID-19 pandemic has subsided, it is crucial that we continue to leverage modern tools and technologies, as they can prove invaluable for patients residing in remote or underserved areas, ensuring better access to quality healthcare. In this cross-sectional survey, we found molecular pathways and biomarkers important in diagnostic and therapeutic processes as well as in the prevention of DR. VEGF is one of the most crucial pro-angiogenic factors, which plays an important role in PDR. Ischemia and hypoxia stimulate VEGF production, and through its many tyrosine kinase receptors, they accelerate the angiogenesis and vascular remodeling in the retina. According to current PDR studies, understanding the complex mechanisms of VEGF signaling pathways is necessary to indicate targets for biological treatment, which can be a milestone in DR therapy. VEGF seems to be an appropriate biomarker, helpful in diagnosing and differentiating the severity of DR. Samples for VEGF level measurements might be taken from blood, vitreous or aqueous fluid, and tears as well. Especially tear VEGF levels might be used as a non-invasive tool to expedite screening programs and assess the severity of DR in patients with diabetes. Using VEGF as a predictive factor of DR development or as a marker of therapeutic management notably in anti-VEGF therapy needs further analysis in dedicated studies. Other factors affecting VEGF levels should be investigated.

ADMA inhibits the activity of NOS, which results in decreased levels of NO and leads to vasoconstriction and endothelial dysfunction. Increased ADMA levels may be considered an early prognostic factor of diabetes complications such as PDR. The use of ADMA as a biomarker may help in early diagnosis, monitoring, and effective therapeutic management of the disease. Reducing ADMA levels in patients with diabetes may be a new therapeutic target to prevent the development of diabetic retinopathy. Endothelin-1 is another factor with an undoubted relationship to diabetic retinopathy. Increased serum and aqueous humor levels are observed in patients with ET-1 elevation dependent on the severity of the progression of the disease. This, juxtaposed with promising results of ET-1 receptor antagonist animal studies, showcases the potential of ET-1 as a possible target for future therapy. It is important to note that miRNAs are not only supposed to be an innovative predictive biomarker and progression indicator in DR but also a potential therapeutic target. Different miRNAs can be found in T1DM and T2DM as well depending on sample type, moreover, some of them differ depending on DR type. The variety of miRNAs and frequently high amounts of particles involved in several pathogenesis pathways can be at the same time the advantage and disadvantage of that prospective novel biomarkers group; hence, miRNAs panels are more adequate than a single biomarker rating. Finally, advanced glycation end products play a significant role in the pathophysiology of diabetic retinopathy causing impairment of the neurovascular units through reactive oxygen species, inflammatory reactions, and cell death pathways. All the above mechanisms play a significant role not only in diabetic retinal disorders, but also other chronic oxidative-based diseases; therefore, a thorough understanding of their properties and mechanisms will allow advances in the diagnosis and treatment of chronic diseases and most importantly diabetic retinopathy. The above factors and signaling pathways can help to create multimodal and highly specified therapies for patients suffering from DR. It is crucial to investigate molecular agents participating in DR pathogenesis. Hopefully, it will provide the ability to inhibit this progressive disease at its early stage.

## 7. Conclusions

DR as a serious complication of DM needs an advanced diagnostic and therapeutic process. We pointed out the vital biomarkers, which can be helpful in DR investigation.

VEGF is an important pro-angiogenic factor in DR progression. Amid all analyzed techniques, tear VEGF level especially seems to be a promising innovative diagnostic method, mainly because of its non-invasiveness, high sensitivity, and specificity. However, studies suggest that VEGF levels should not be considered as a predictive factor of DR development.

ADMA has significant clinical relevance, and it seems to be reasonable to investigate all possible factors responsible for its level in the organism. Elevated ADMA levels are associated with endothelial dysfunction, oxidative stress, and may contribute to the development and progression of DR. Further research is warranted to explore medications aimed at lowering ADMA, and additional clinical studies are essential to assess whether reducing ADMA levels can effectively decelerate the progression of diabetic microvascular complications and yield improved prognoses.

MiRNAs are promising biomarkers, however, their numerousness may be their disadvantage according to the number of results that would be needed to obtain as well as an advantage. Thanks to this there is a possibility to distinguish various miRNAs panels accurate for specific DM types.

ET-1 is a multifaceted hormone with a crucial role in vascular regulation and endothelial dysfunction, particularly in the context of DR. Elevated ET-1 levels in diabetic patients, especially those with advanced DR, suggest its involvement in the pathogenesis of this condition. While promising findings from animal studies and early human trials indicate the potential therapeutic value of ET-1 receptor antagonists in mitigating DR-related microvascular changes and neurodegeneration, further research is needed to validate their efficacy and safety for clinical use.

Using the biochemical properties of AGEs, which play a significant role in the pathogenesis of DR, as a biomarker is a promising direction in the diagnosis of diabetic retinopathy. A method based on skin autofluorescence testing would provide an easy, accessible, non-invasive, and relatively rapid way to identify patients at risk of developing diabetic retinopathy.

## 8. Limitations

Our study has several limitations. The results of some of the publications used in the process of writing the above paper present outcomes based on small groups of patients or homogeneous populations, which raises the need for studies on groups both more numerous and diverse in age, gender, and ethnicity. There is a lack of studies focused on a practical clinical approach rather than on the biochemical characteristics of the molecules presented. Further systematic review and meta-analysis should be conducted to confirm the diagnostic value of the above biomarkers.

## Figures and Tables

**Table 1:** VEGF receptors and their ligands.

Receptor	VEGFR1	VEGFR2	VEGFR3
VEGF variant	VEGF-A, -B, and PGF	VEGF-A, -C, and -D	VEGF-C and -D

## Data Availability

Data sharing is not applicable as no datasets were generated or analyzed during the current study.

## References

[1] Tan T.E., Wong T.Y. (2023). Diabetic retinopathy: Looking forward to 2030. Front. Endocrinol..

[2] International Diabetes Federation International Diabetes Federation—Facts & Figures. Idf.org. Published 12 September 2021. https://www.idf.org/aboutdiabetes/what-is-diabetes/facts-figures.html.

[3] Cheung N., Mitchell P., Wong T.Y. (2010). Diabetic retinopathy. Lancet.

[4] Modjtahedi B.S., Wu J., Luong T.Q., Gandhi N.K., Fong D.S., Chen W. (2021). Severity of Diabetic Retinopathy and the Risk of Future Cerebrovascular Disease, Cardiovascular Disease, and All-Cause Mortality. Ophthalmology.

[5] Teo Z.L., Tham Y.C., Yu M., Chee M.L., Rim T.H., Cheung N., Bikbov M.M., Wang Y.X., Tang Y., Lu Y. (2021). Global Prevalence of Diabetic Retinopathy and Projection of Burden through 2045: Systematic Review and Meta-analysis. Ophthalmology.

[6] Ahmed I., Liu T.Y.A. (2021). The Impact of COVID-19 on Diabetic Retinopathy Monitoring and Treatment. Curr. Diabetes Rep..

[7] Romero-Aroca P., Baget-Bernaldiz M., Sagarra R., Hervás E., Blasco R., Molina J., Moreno E.F., Garcia-Curto E. (2022). Impact of the COVID-19 Pandemic on the Metabolic Control of Diabetic Patients in Diabetic Retinopathy and Its Screening. J. Clin. Med..

[8] Centers for Disease Control and Prevention Vision & Eye Health Surveillance System (VEHSS), Vision Health Initiative. https://www.cdc.gov/visionhealth/vehss/project/index.html.

[9] Galiero R., Pafundi P.C., Nevola R., Rinalida L. (2020). The Importance of Telemedicine during COVID-19 Pandemic: A Focus on Diabetic Retinopathy. J. Diabetes Res..

[10] Robles R., Patel N., Neag E., Mittal A., Markatia Z., Ameli K., Lin B. (2023). A Systematic Review of Digital Ophthalmoscopes in Medicine. Clin. Ophthalmol..

[11] Sasso F.C., Pafundi P.C., Gelso A., Bono C. (2019). Telemedicine for screening diabetic retinopathy: The NO BLIND Italian multicenter study. Diabetes Metab. Res. Rev..

[12] Lin K.Y., Hsih W.H., Lin Y.B., Wen C.Y., Chang T.J. (2021). Update in the epidemiology, risk factors, screening, and treatment of diabetic retinopathy. J. Diabetes Investig..

[13] Chew E.Y., Davis M.D., Danis R.P., Locato J.F. (2014). The effects of medical management on the progression of diabetic retinopathy in persons with type 2 diabetes: The Action to Control Cardiovascular Risk in Diabetes (ACCORD) Eye Study. Ophthalmology..

[14] Action to Control Cardiovascular Risk in Diabetes Follow-On (ACCORDION) Eye Study Group, The Action to Control Cardiovascular Risk in Diabetes Follow-On (ACCORDION) Study Group (2016). Persistent effects of intensive glycemic control on retinopathy in type 2 diabetes in the Action to Control Cardiovascular Risk in Diabetes (ACCORD) follow-on study. Diabetes Care.

[15] Kaštelan S., Tomić M., Gverović Antunica A., Ljubić S., Salopek Rabatić J., Karabatić M. (2013). Body mass index: A risk factor for retinopathy in type 2 diabetic patients. Mediat. Inflamm..

[16] Chang Y.C., Wu W.C. (2013). Dyslipidemia and diabetic retinopathy. Rev. Diabet. Stud..

[17] Kohner E.M., Aldington S.J., Stratton I.M., Manley S.E., Holman R.R., Matthews D.R., Turner R.C. (1998). United Kingdom Prospective Diabetes Study 30. Diabetic retinopathy at diagnosis of non-insulin-dependent diabetes mellitus and associated risk factors. Arch. Ophthalmol..

[18] Klein R., Sharrett A.R., Klein B.E., Moss S.E., Folsom A.R., Wong T.Y., Brancati F.L., Hubbard L.D., Couper D., ARIC Group (2002). The association of atherosclerosis, vascular risk factors, and retinopathy in adults with diabetes: The Atherosclerosis Risk in Communities study. Ophthalmology.

[19] Klein R., Marino E.K., Kuller L.H., Polak J.F., Tracy R.P., Gottdiener J.S., Burke G.L., Hubbard L.D., Boineau R. (2002). The relation of atherosclerotic cardiovascular disease to retinopathy in people with diabetes in the Cardiovascular Health Study. Br. J. Ophthalmol..

[20] van Leiden H.A., Dekker J.M., Moll A.C., Nijpels G., Heine R.J., Bouter L.M., Stehouwer C.D., Polak B.C. (2002). Blood pressure, lipids, and obesity are associated with retinopathy: The Hoorn study. Diabetes Care.

[21] Vujosevic S., Aldington S.J., Silva P., Peto P. (2020). Screening for diabetic retinopathy: New perspectives and challenges. Lancet Diabetes Endocrinol..

[22] Cai X., Chen Y., Yang W., Gao X., Han X., Ji L. (2018). The association of smoking and risk of diabetic retinopathy in patients with type 1 and type 2 diabetes: A meta-analysis. Endocrine.

[23] Rasmussen K.L., Laugesen C.S., Ringholm L., Vestgaard M., Damm P., Mathiesen E.R. (2010). Progression of diabetic retinopathy during pregnancy in women with type 2 diabetes. Diabetologia.

[24] Rubsam A., Parikh S., Fort P.E. (2018). Role of Inflammation in Diabetic Retinopathy. Int. J. Mol. Sci..

[25] Simo R., Stitt A.W., Gardner T.W. (2018). Neurodegeneration in diabetic retinopathy: Does it really matter?. Diabetologia.

[26] Ansari P., Tabasumma N., Snigdha N.N., Siam N.H., Panduru R.V.N.R.S., Azam S., Hannan J.M.A., Abdel-Wahab Y.H.A. (2022). Diabetic Retinopathy: An Overview on Mechanisms, Pathophysiology and Pharmacotherapy. Diabetology.

[27] Kowluru R.A., Santos J.M., Mishra M. (2013). Epigenetic modifications and diabetic retinopathy. Biomed. Res. Int..

[28] Kollias A.N., Ulbig M.W. (2010). Diabetic retinopathy: Early diagnosis and effective treatment. Dtsch. Arztebl. Int..

[29] Carmeliet P. (2000). Mechanisms of angiogenesis and arteriogenesis. Nat. Med..

[30] Ishida S., Usui T., Yamashiro K., Kaji Y., Ahmed E., Carrasquillo K.G., Amano S., Hida T., Oguchi Y., Adamis A.P. (2003). VEGF164 is proinflammatory in the diabetic retina. Investig. Ophthalmol. Vis. Sci..

[31] Williams B., Baker A.Q., Gallacher B., Lodwick D. (1995). Angiotensin II increases vascular permeability factor gene expression by human vascular smooth muscle cells. Hypertension.

[32] Viswanath K., McGavin D.D. (2003). Diabetic retinopathy: Clinical findings and management. Community Eye Health.

[33] Roy S., Kim D. (2021). Retinal capillary basement membrane thickening: Role in the pathogenesis of diabetic retinopathy. Prog. Retin. Eye Res..

[34] Kern T.S., Huang S.S. (2010). Vascular damage in diabetic retinopathy. Ocul. Dis. Mech. Manag..

[35] Bakri S.J., Kaiser P.K., Huang D., Kaiser P.K., Lowder C.Y., Elias I., Traboulsi E.I. (2006). Chapter 21—Diabetic Retinopathy. Retinal Imaging.

[36] Aronson J.K., Ferner R.E. (2017). Biomarkers—A General Review. Curr. Protoc. Pharmacol..

[37] Nawaz I.M., Rezzola S., Cancarini A., Russo A., Costagliola C., Semeraro F., Presta M. (2019). Human vitreous in proliferative diabetic retinopathy: Characterization and translational implications. Prog. Retin. Eye Res..

[38] Homsi J., Daud A.I. (2007). Spectrum of activity and mechanism of action of VEGF/PDGF inhibitors. Cancer Control.

[39] Shibuya M. (2005). Vascular endothelial growth factor receptor-2: Its unique signaling and specific ligand, VEGF-E. Cancer Sci..

[40] Arrigo A., Aragona E., Bandello F. (2022). VEGF-targeting drugs for the treatment of retinal neovascularization in diabetic retinopathy. Ann. Med..

[41] Holmes D.I., Zachary I. (2005). The vascular endothelial growth factor (VEGF) family: Angiogenic factors in health and disease. Genome Biol..

[42] Gupta N., Mansoor S., Sharma A., Sapkal A., Sheth J., Falatoonzadeh P., Kuppermann B., Kenney M. (2013). Diabetic retinopathy and VEGF. Open Ophthalmol. J..

[43] Ferrara N. (2004). Vascular Endothelial Growth Factor: Basic Science and Clinical Progress. Endocr. Rev..

[44] Stuttfeld E., Ballmer-Hofer K. (2009). Structure and function of VEGF receptors. IUBMB Life.

[45] Clauss M. (2000). Molecular Biology of the VEGF and the VEGF Receptor Family. Semin. Thromb. Hemost..

[46] Wang X., Bove A.M., Simone G., Ma B. (2020). Molecular Bases of VEGFR-2-Mediated Physiological Function and Pathological Role. Front. Cell Dev. Biol..

[47] Claesson-Welsh L. (2016). VEGF receptor signal transduction—A brief update. Vasc. Pharmacol..

[48] Carmeliet P., Ferreira V., Breier G., Harpal K. (1996). Abnormal blood vessel development and lethality in embryos lacking a single VEGF allele. Nature.

[49] Bucolo C., Barbieri A., Vigano I., Band F. (2021). Short-and Long-Term Expression of Vegf: A Temporal Regulation of a Key Factor in Diabetic Retinopathy. Front. Pharmacol..

[50] Hirano T., Toriyama Y., Iesato Y., Imai A., Murata T. (2018). Changes in plasma vascular endothelial growth factor level after intravitreal injection of Bevacizumab, Aflibercept, or Ranibizumab for diabetic macular edema. Retina.

[51] Wu F., Phone A., Lamy R., Chen Y. (2020). Correlation of Aqueous, Vitreous, and Plasma Cytokine Levels in Patients with Proliferative Diabetic Retinopathy. Investig. Ophthalmol. Vis. Sci..

[52] Midena E., Frizziero L., Midena G., Pilotto T. (2021). Intraocular fluid biomarkers (liquid biopsy) in human diabetic retinopathy. Graefes Arch. Clin. Exp. Ophthalmol..

[53] Bonfiglio V., Platania C.B.M., Lazzara F., Conti F., Pizzo C., Reibaldi M., Russo A., Fallico M., Ortisi E., Pignatelli F. (2020). TGF-β Serum Levels in Diabetic Retinopathy Patients and the Role of Anti-VEGF Therapy. Int. J. Mol. Sci..

[54] Nalini M., Raghavulu B.V., Annapurna A., Chandl V. (2017). Correlation of various serum biomarkers with the severity of diabetic retinopathy. Diabetes Metab. Syndr. Clin. Res. Rev..

[55] Nakhleh E., Abu Y., Nafez M., Abu T., Ala MAbojaradeh A.S., Al-Akily E.M., Abdo L., Emoush O. (2020). Relationship between Serum Vascular Endothelial Growth Factor Levels and Stages of Diabetic Retinopathy and Other Biomarkers. J. Ophthalmol..

[56] Ahuja S., Saxena S., Akduman L., Khanna V. (2019). Serum vascular endothelial growth factor is a biomolecular biomarker of severity of diabetic retinopathy. Int. J. Retin. Vitr..

[57] Ang W.J., Zunaina E., Norfadzillah A.J., Lewin A.S. (2019). Evaluation of vascular endothelial growth factor levels in tears and serum among diabetic patients. PLoS ONE.

[58] Kaštelan S., Orešković I., Bišćan F., Kaštelan H., Gverović Antunica A. (2020). Inflammatory and angiogenic biomarkers in diabetic retinopathy. Biochem. Medica.

[59] Majidreza S., Alizadeh M., Saeid A. (2019). The tear VEGF and IGFBP3 in healthy and diabetic retinopathy. Int. J. Diabetes Dev. Ctries..

[60] Mei C., Pan L., Xu W., Li Z. (2021). An ultrasensitive reusable aptasensor for noninvasive diabetic retinopathy diagnosis target on tear biomarker. Sens. Actuators B Chem..

[61] Wang J.Y., Kwon J.S., Hsu S.M., Chuang H.S. (2020). Sensitive tear screening of diabetic retinopathy with dual biomarkers enabled using a rapid electrokinetic patterning platform. Lab A Chip.

[62] Hashimoto Y., Okada A., Matsui H., Obata R. (2023). Recent trends in anti-vascular endothelial growth factor intravitreal injections: A large claims database study in Japan. Jpn. J. Ophthalmol..

[63] Grillo M.A., Colombatto S. (2004). Arginine revisited: Minireview article. Amino Acids.

[64] Endemann D.H., Schiffrin E.L. (2004). Endothelial dysfunction. J. Am. Soc. Nephrol..

[65] Yonem A., Duran C., Unal M., Ipcioglu O.M., Ozcan O. (2009). Plasma apelin and asymmetric dimethylarginine levels in type 2 diabetic patients with diabetic retinopathy. Diabetes Res. Clin. Pract..

[66] Narayanan P.S., Rojas M., Suwanpradid J., Toque H.A., Caldwell W.R., Caldwell R.B. (2013). Arginase in retinopathy. Prog. Retin. Eye Res..

[67] Sena C.M., Pereira A.M., Seica R. (2013). Endothelial dysfunction—A major mediator of diabetic vascular disease. Biochim. Biophys. Acta.

[68] Forstermann U., Closs E.I., Pollock J.S., Nakane M., Schwarz P., Gath I., Kleinert H. (1994). Nitric oxide synthase isozymes. Characterization, purification, molecular cloning, and functions. Hypertension.

[69] Toutouzas K., Riga M., Stefanadi E., Stefanadis C. (2008). Asymmetric dimethylarginine (ADMA) and other endogenous nitric oxide synthase (NOS) inhibitors as an important cause of vascular insulin resistance. Horm. Metab. Res..

[70] Bode-Boger S.M., Scalera F., Martens-Lobenhoffer J. (2005). Asymmetric dimethylarginine (ADMA) accelerates cell senescence. Vasc. Med..

[71] Sirman Y.V., Savytskyi I.V. (2019). Study of endothelial dysfunction and asymmetric dimethylarginine levels. J. Educ. Health Sport.

[72] Leiper J.M., Vallance P. (2006). The synthesis and metabolism of asymmetric dimethylarginine (ADMA). Eur. J. Clin. Pharmacol..

[73] Morris S.M. (2016). Arginine metabolism revisited. J. Nutr..

[74] Trocha M., Merwid-Lad A., Szuba A., Sozanski T., Magdalan J., Szelag A. (2010). Asymmetric dimethylarginine synthesis and degradation under physiological and pathological conditions. Adv. Clin. Exp. Med..

[75] Vallance P., Leone A., Calver A., Collier J., Moncada S. (1992). Accumulation of an endogenous inhibitor of nitric oxide synthesis in chronic renal failure. Lancet.

[76] Sydow K., Munzel T. (2003). ADMA and oxidative stress. Atheroscler. Suppl..

[77] Cardounel A.J., Cui H., Samouilov A., Johnson W., Kearns P., Tsai A.-L., Berka V., Zweier J.L. (2007). Evidence for the pathophysiological role of endogenous methylarginines in regulation of endothelial NO production and vascular function. J. Biol. Chem..

[78] Jian Q., Wu Y., Zhang F. (2022). Metabolomics in diabetic retinopathy: From potential biomarkers to molecular basis of oxidative stress. Cells.

[79] Peters K.S., Rivera E., Warden C., Harlow P.A., Mitchell S.L., Calcutt M.W., Samuels D.C., Brantley M.A. (2022). Plasma arginine and citrulline are elevated in diabetic retinopathy. Am. J. Ophthalmol..

[80] Sumarriva K., Uppal K., Ma C., Herren D.J., Wang Y., Chocron I.M., Warden C., Mitchell S.L., Burgess G.L., Goodale M.P. (2019). Arginine and carnitine metabolites are altered in diabetic retinopathy. Investig. Ophthalmol. Vis. Sci..

[81] Dag U., Caglayan M., Alakus M.F., Oncul H. (2023). The relationship between reduced choroidal thickness due to high plasma asymmetrical dimethylarginine level and increased severity of diabetic retinopathy. Arq. Bras. Oftalmol..

[82] Lamprou S., Koletsos N., Mintziori G., Anyfanti P., Trakatelli C., Kotsis V., Gkaliagkousi E., Triantafyllou A. (2023). Microvascular and endothelial dysfunction in prediabetes. Life.

[83] Krasnicki P., Proniewska-Skretek E., Dmuchowska D.A., Dobrzycki S., Mariak Z. (2009). Asymmetric dimethylarginine (ADMA) as a marker of blood flow disturbances in ocular circulation in patients with type 2 diabetes and coronary artery disease. Mag. Lek. Okulisty.

[84] Tousoulis D., Kampoli A.-M., Stefanadis C. (2012). Diabetes mellitus and vascular endothelial dysfunction: Current perspectives. Curr. Vasc. Pharmacol..

[85] Stepien E., Szuscik I., Tokarz A., Enguita F.J., Solnica B., Zurakowski A., Malecki M. (2014). The role of microparticles in pathomechanisms of diabetic retinopathy—Analysis of intercellular communication mechanisms in endothelial aging. Case control study in patients with metabolic syndrome, diabetes type 1 and type 2. J. Med. Sci..

[86] Huang C.-Y., Zhou T., Li G., Li M.-Y., Xiong X.-M., Wu M.-T., Jiang J.-L. (2019). Asymmetric dimethylarginine aggravates blood-retinal barrier breakdown of diabetic retinopathy via inhibition of intercellular communication in retinal pericytes. Amino Acids.

[87] Liu J., Li C., Chen W., He K., Ma H., Ma B., Zhao P., Tian L. (2019). Relationship between serum asymmetric dimethylarginine level and microvascular. Bio. Med. Res. Int..

[88] Alpay A., Ozcan O., Ugurbas S.C., Ugurbas S.H. (2019). Investigated of vitreous and serum asymmetric dimethylarginine levels in diabetic. Res. Sq..

[89] Sugai M., Ohta A., Ogata Y., Nakanishi M., Ueno S., Kawata T., Saito N., Tanaka Y. (2007). Asymmetric dimethylarginine (ADMA) in the aqueous humor of diabetic patients. Endocr. J..

[90] Abhary S., Kasmeridis N., Burdon K.P., Kuot A., Whiting M.J., Yew W.P., Petrovsky N., Craig J.E. (2009). Diabetic retinopathy is associated with elevated serum asymmetric and symmetric dimethylarginines. Diabetes Care.

[91] Eliana F., Suwondo P., Makmun L.H., Harbuwono D.S. (2011). ADMA as a marker of endothelial dysfunction in prediabetic women. Acta Medica Indones..

[92] Du M.-R., Yan L., Li N.-S., Wang Y.-J., Zhou T., Jiang J.-L. (2018). Asymmetric dimethylarginine contributes to retinal neovascularization of diabetic retinopathy through EphrinB2 pathway. Vasc. Pharmacol..

[93] Yun J.H., Kim J.-M., Jeon H.J., Oh T., Choi H.J., Kim B.-J. (2020). Metabolomics profiles associated with diabetic retinopathy in type 2 diabetes patients. PLoS ONE.

[94] Malecki M.T., Undas A., Cyganek K., Mirkiewicz-Sieradzka B., Wolkow P., Osmenda G., Walus-Miarka M., Guzik T.J., Sieradzki J. (2007). Plasma asymmetric dimethylarginine (ADMA) is associated with retinopathy in type 2 diabetes. Diabetes Care.

[95] Hernandes C., Porta M., Bandello F., Grauslund J., Harding S.P., Aldington S.J., Egan C., Frydkjaer-Olsen U., Garcia-Arumi J., Gibson J. (2020). The usefulness of serum biomarkers in the early stages of diabetic retinopathy: Results of the EUROCONDOR clinical trial. J. Clin. Med..

[96] Aydogan S., Dilli D., Kabatas E.U., Akduman H., Sah Ipek M., Oguz B., Atlas N., Zenciroglu A. (2022). The serum levels of asymmetric dimethylarginine, vascular endothelial growth factor, and insulin-like growth factor-1 in preterms with retinopathy of prematurity. Fetal Pediatr. Pathol..

[97] Wieczor R., Wieczor A.M., Kulwas A., Rosc D. (2021). ADMA (asymmetric dimethylarginine) and angiogenic potential in patients with type 2 diabetes and prediabetes. Exp. Biol. Med..

[98] Sibal L., Agarwal S.C., Home P.D., Boger R.H. (2010). The role of asymmetric dimethylarginine (ADMA) in endothelial dysfunction and cardiovascular disease. Curr. Cardiol. Rev..

[99] Jaroszynski A.J., Jaroszynska A., Wysokinski A. (2012). Asymmetric dimethylarginine—The link between hart and kidney diseases. Chor. Serca I Naczyń.

[100] Celik M., Cerrah S., Arabul M., Akalin A. (2014). Relation of asymmetric dimethylarginine levels to macrovascular disease and inflammation markers in type 2 diabetic patients. J. Diabetes Res..

[101] Kawata T., Daimon M., Hasegawa R., Teramoto K., Toyoda T., Sekine T., Yamamoto K., Uchida D., Himi T., Yoshida K. (2009). Effect of angiotensin-converting enzyme inhibitor on serum asymmetric dimethylarginine and coronary circulation in patients with type 2 diabetes mellitus. Int. J. Cardiol..

[102] Guo X., Xing Y., Jin W. (2023). Role of ADMA in the pathogenesis of microvascular complications in type 2 diabetes mellitus. Front. Endocrinol..

[103] O’Brien J., Hayder H., Zayed Y., Peng C. (2018). Overview of MicroRNA Biogenesis, Mechanisms of Actions, and Circulation. Front. Endocrinol..

[104] Ha M., Kim V. (2014). Regulation of microRNA biogenesis. Nat. Rev. Mol. Cell Biol..

[105] Glinge C., Clauss S., Boddum K., Jabbari R., Jabbari J., Risgaard B., Tomsits P., Hildebrand B., Kaab S., Wakili R. (2017). Stability of Circulating Blood-Based MicroRNAs—Pre-Analytic Methodological Considerations. PLoS ONE.

[106] Karbasforooshan H., Karimi G. (2018). The role of SIRT1 in diabetic retinopathy. Biomed. Pharmacother..

[107] Chang X., Zhu G., Cai Z., Wang Y., Lian R., Tang X., Ma C., Fu S. (2021). miRNA, lncRNA and circRNA: Targeted Molecules Full of Therapeutic Prospects in the Development of Diabetic Retinopathy. Front. Endocrinol..

[108] Ji Q., Han J., Wang L., Liu J., Dong Y., Zhu K., Shi L. (2020). MicroRNA-34a promotes apoptosis of retinal vascular endothelial cells by targeting SIRT1 in rats with diabetic retinopathy. Cell Cycle.

[109] Shan L., Zhang H., Han Y., Kuang R. (2022). Expression and mechanism of microRNA 195 in diabetic retinopathy. Endocr. J..

[110] Yin C., Lin X., Sun Y., Ji X. (2020). Dysregulation of miR210 is involved in the development of diabetic retinopathy and serves a regulatory role in retinal vascular endothelial cell proliferation. Eur. J. Med. Res..

[111] Pan Q., Gao Z., Zhu C., Peng Z., Song M., Li L. (2020). Overexpression of histone deacetylase SIRT1 exerts an antiangiogenic role in diabetic retinopathy via miR-20a elevation and YAP/HIF1α/VEGFA depletion. Am. J. Physiol. Endocrinol. Metab..

[112] Qin B., Liu J., Liu S., Li B., Ren J. (2016). MiR-20b targets AKT3 and modulates vascular endothelial growth factor-mediated changes in diabetic retinopathy. Acta Biochim. Biophys. Sin..

[113] Maisto R., Trotta M.C., Petrillo F., Izzo S., Cuomo G., Alfano R., Hermenean A., Barcia J.M., Galdiero M., Platania C.B.M. (2020). Resolvin D1 Modulates the Intracellular VEGF-Related miRNAs of Retinal Photoreceptors Challenged With High Glucose. Front. Pharmacol..

[114] Duan P., Chen S., Zeng Y., Xu H., Liu Y. (2020). Osteopontin Upregulates Col IV Expression by Repressing miR-29a in Human Retinal Capillary Endothelial Cells. Mol. Ther. Nucleic Acids.

[115] Someya H., Ito M., Nishio Y., Sato T., Harimoto K., Takeuchi M. (2022). Osteopontin-induced vascular hyperpermeability through tight junction disruption in diabetic retina. Exp. Eye Res..

[116] Wang J., Yao Y., Wang K., Chu T. (2020). MicroRNA-148a-3p alleviates high glucose-induced diabetic retinopathy by targeting TGFB2 and FGF2. Acta Diabetol..

[117] Liu H.N., Cao N.J., Li X., Qian W., Chen X.L. (2018). Serum microRNA-211 as a biomarker for diabetic retinopathy via modulating Sirtuin 1. Biochem. Biophys. Res. Commun..

[118] Miao C., Chang J., Zhang G., Fang Y. (2018). MicroRNAs in type 1 diabetes: New research progress and potential directions. Biochem. Cell Biol..

[119] Margaritis K., Margioula-Siarkou G., Giza S., Kotanidou E.P., Tsinopoulou V.R., Christoforidis A., Galli-Tsinopoulou A. (2021). Micro-RNA Implications in Type-1 Diabetes Mellitus: A Review of Literature. Int. J. Mol. Sci..

[120] Li E.H., Huang Q.Z., Li G.C., Xiang Z.Y., Zhang X. (2017). Effects of miRNA-200b on the development of diabetic retinopathy by targeting VEGFA gene. Biosci. Rep..

[121] Liang Z., Gao K.P., Wang Y.X., Liu Z.C., Tian L., Yang X.Z., Ding J.Y., Wu W.T., Yang W.H., Li Y.L. (2018). RNA sequencing identified specific circulating miRNA biomarkers for early detection of diabetes retinopathy. Am. J. Physiol. Endocrinol. Metab..

[122] Santovito D., Toto L., De Nardis V., Ces D. (2021). Plasma microRNA signature associated with retinopathy in patients with type 2 diabetes. Sci Rep..

[123] McArthur K., Feng B., Wu Y., Chen S., Chakrabarti S. (2011). MicroRNA-200b regulates vascular endothelial growth factor-mediated alterations in diabetic retinopathy. Diabetes.

[124] Yang Y., Yue W., Wang N., Wang Z., Li B., Zeng J., Yoshida S., Ding C., Zhou Y. (2022). Altered Expressions of Transfer RNA-Derived Small RNAs and microRNAs in the Vitreous Humor of Proliferative Diabetic Retinopathy. Front. Endocrinol..

[125] Kot A., Kaczmarek R. (2022). Exosomal miRNA Profiling in Vitreous Humor in Proliferative Diabetic Retinopathy. Cells.

[126] Guo J., Zhou P., Liu Z., Dai F., Pan M., An G., Han J., Du L., Jin X. (2021). The Aflibercept-Induced MicroRNA Profile in the Vitreous of Proliferative Diabetic Retinopathy Patients Detected by Next-Generation Sequencing. Front. Pharmacol..

[127] Saleh A.A., El-Hefnawy S.M., Kasemy Z.A., Alhagaa A.A., Nooh M.Z., Arafat E.S. (2022). Mi-RNA-93 and Mi-RNA-152 in the Diagnosis of Type 2 Diabetes and Diabetic Retinopathy. Br. J. Biomed. Sci..

[128] Zampetaki A., Kiechl S., Drozdov I., Willeit P., Mayr U., Prokopi M., Mayr A., Weger S., Oberhollenzer F., Bonora E. (2010). Plasma microRNA profiling reveals loss of endothelial miR-126 and other microRNAs in type 2 diabetes. Circ. Res..

[129] Ko G.Y., Yu F., Bayless K.J., Ko M.L. (2022). MicroRNA-150 (miR-150) and Diabetic Retinopathy: Is miR-150 Only a Biomarker or Does It Contribute to Disease Progression?. Int. J. Mol. Sci..

[130] Zhou H., Peng C., Huang D.S., Liu L., Guan P. (2020). microRNA Expression Profiling Based on Microarray Approach in Human Diabetic Retinopathy: A Systematic Review and Meta-Analysis. DNA Cell Biol..

[131] Ma L., Wen Y., Li Z., Wu N., Wang Q. (2022). Circulating MicroRNAs as Potential Diagnostic Biomarkers for Diabetic Retinopathy: A Meta-Analysis. Front. Endocrinol..

[132] Jenkins H.N., Rivera-Gonzalez O., Gibert Y., Speed J.S. (2020). Endothelin-1 in the pathophysiology of obesity and insulin resistance. Obes. Rev..

[133] Ergul A. (2011). Endothelin-1 and diabetic complications: Focus on the vasculature. Pharmacol. Res..

[134] Stow L.R., Jacobs M.E., Wingo C.S., Cain B.D. (2010). Endothelin-1 gene regulation. FASEB J..

[135] Kostov K. (2021). The causal relationship between endothelin-1 and hypertension: Focusing on endothelial dysfunction, arterial stiffness, vascular remodeling, and Blood Pressure Regulation. Life.

[136] Abman S.H. (2009). Role of endothelin receptor antagonists in the treatment of pulmonary arterial hypertension. Annu. Rev. Med..

[137] Cheung S.S., Leung J.W., Lam A.K., Acy L. (2011). Selective over-expression of endothelin-1 in endothelial cells exacerbates inner retinal edema and neuronal death in ischemic retina. PLoS ONE.

[138] Chen Y.-L., Rosa R.H., Kuo L., Hein T.W. (2020). Hyperglycemia augments endothelin-1–induced constriction of human retinal venules. Transl. Vis. Sci. Technol..

[139] Chang W., Lajko M., Fawzi A.A. (2018). Endothelin-1 is associated with fibrosis in proliferative diabetic retinopathy membranes. PLoS ONE.

[140] Kang H.M., Hasanuzzaman M., Kim S.W., Koh H.J., Lee S.C. (2022). Elevated aqueous endothelin-1 concentrations in advanced diabetic retinopathy. PLoS ONE.

[141] Khuu L.-A., Tayyari F., Sivak J.M., Singer S. (2017). Aqueous humor endothelin-1 and total retinal blood flow in patients with non-proliferative diabetic retinopathy. Eye.

[142] Ottosson-Seeberger A., Lundberg J.M., Alvestrand A., Ahlborg G. (1997). Exogenous endothelin-1 causes peripheral insulin resistance in healthy humans. Acta Physiol. Scand..

[143] Anfossi G., Russo I., Doronzo G., Trovati M. (2007). Relevance of the vascular effects of insulin in the rationale of its therapeutical use. Cardiovasc. Hematol. Disord. Drug Targets.

[144] Wang Z., Yadav A.S., Leskova W., Harris N.R. (2010). Attenuation of streptozotocin-induced microvascular changes in the mouse retina with the endothelin receptor A antagonist atrasentan. Exp. Eye Res..

[145] Chou J.C., Rollins S.D., Ye M., Batlle D., Fawzi A.A. (2014). Endothelin receptor-a antagonist attenuates retinal vascular and neuroretinal pathology in diabetic mice. Investig. Opthalmology Vis. Sci..

[146] Alrashdi S.F., Deliyanti D., Wilkinson-Berka J.L. (2018). Intravitreal administration of endothelin type A receptor or endothelin type B receptor antagonists attenuates hypertensive and diabetic retinopathy in rats. Exp. Eye Res..

[147] Bogdanov P., Simo-Servat O., Sampedro J., Garcia M. (2018). Topical administration of Bosentan prevents retinal neurodegeneration in experimental diabetes. Int. J. Mol. Sci..

[148] Shen C.Y., Lu C.H., Wu C.H., Li K.J. (2020). The Development of Maillard Reaction, and Advanced Glycation End Product (AGE)-Receptor for AGE (RAGE) Signaling Inhibitors as Novel Therapeutic Strategies for Patients with AGE-Related Diseases. Molecules.

[149] Ruiz H.H., Ramasamy R., Schmidt A.M. (2020). Advanced Glycation End Products: Building on the Concept of the “Common Soil” in Metabolic Disease. Endocrinology.

[150] Reddy V.P., Aryal P., Darkwah E.K. (2022). Advanced Glycation End Products in Health and Disease. Microorganisms.

[151] Khalid M., Petroianu G., Adem A. (2022). Advanced Glycation End Products and Diabetes Mellitus: Mechanisms and Perspectives. Biomolecules.

[152] Mao L., Yin R., Yang L., Zhao D. (2022). Role of advanced glycation end products on vascular smooth muscle cells under diabetic atherosclerosis. Front. Endocrinol..

[153] Vlassara H., Bucala R., Striker L. (1988). Pathogenic effects of advanced glycosylation: Biochemical, biologic, and clinical implications for diabetes and aging. Lab. Investig..

[154] Zhang Q., Wang Y., Fu L. (2020). Dietary advanced glycation end-products: Perspectives linking food processing with health implications. Compr. Rev. Food Sci. Food Saf..

[155] Vistoli G., De Maddis D., Cipak A., Zarkovic N., Carini M., Aldini G. (2013). Advanced glycoxidation and lipoxidation end products (AGEs and ALEs): An overview of their mechanisms of formation. Free Radic. Res..

[156] Uribarri J., Woodruff S., Goodman S., Cai W. (2010). Advanced glycation end products in foods and a practical guide to their reduction in the diet. J. Am. Diet. Assoc..

[157] Garay-Sevilla M.E., Rojas A., Portero-Otin M., Uribarri J. (2021). Dietary AGEs as Exogenous Boosters of Inflammation. Nutrients.

[158] Gill V., Kumar V., Singh K., Kumar A., Kim J.J. (2019). Advanced Glycation End Products (AGEs) May Be a Striking Link Between Modern Diet and Health. Biomolecules.

[159] Zawada A., Machowiak A., Rychter A.M., Rata A.E. (2022). Accumulation of Advanced Glycation End-Products in the Body and Dietary Habits. Nutrients.

[160] Perrone A., Giovino A., Benny J., Martinelli F. (2020). Advanced Glycation End Products (AGEs): Biochemistry, Signaling, Analytical Methods, and Epigenetic Effects. Oxid. Med. Cell. Longev..

[161] Oshitari T. (2023). Advanced Glycation End-Products and Diabetic Neuropathy of the Retina. Int. J. Mol. Sci..

[162] Stitt A.W. (2010). AGEs and diabetic retinopathy. Investig. Ophthalmol. Vis. Sci..

[163] Mokini Z., Marcovecchio M.L., Chiarelli F. (2010). Molecular pathology of oxidative stress in diabetic angiopathy: Role of mitochondrial and cellular pathways. Diabetes Res. Clin. Pract..

[164] Safi S.Z., Qvist R., Kumar S., Batumalaie K., Ismail I.S. (2014). Molecular mechanisms of diabetic retinopathy, general preventive strategies, and novel therapeutic targets. Biomed. Res. Int..

[165] Chung Y.R., Choi J.A., Koh J.Y., Yoon Y.H. (2017). Ursodeoxycholic Acid Attenuates Endoplasmic Reticulum Stress-Related Retinal Pericyte Loss in Streptozotocin-Induced Diabetic Mice. J. Diabetes. Res..

[166] Stirban A., Heinemann L. (2014). Skin Autofluorescence—A Non-invasive Measurement for Assessing Cardiovascular Risk and Risk of Diabetes. Eur. Endocrinol..

[167] Vlassara H., Uribarri J. (2014). Advanced glycation end products (AGE) and diabetes: Cause, effect, or both?. Curr. Diab. Rep..

[168] Meerwaldt R., Links T., Zeebregts C., Tio R., Smit O. (2008). The clinical relevance of assessing advanced glycation endproducts accumulation in diabetes. Cardiovasc. Diabetol..

[169] Meerwaldt R., Graaff R., Oomen P.H.N., Jagger J.J. (2004). Simple non-invasive assessment of advanced glycation endproduct accumulation. Diabetologia.

[170] Gerrits E.G., Lutgers H.L., Kleefstra N., Gano N.O. (2008). Skin autofluorescence: A tool to identify type 2 diabetic patients at risk for developing microvascular complications. Diabetes Care.

[171] Osawa S., Katakami N., Sato I., Sakamoto F. (2018). Skin autofluorescence is associated with vascular complications in patients with type 2 diabetes. J. Diabetes Complicat..

